# Sensitivity to Mobile Phone Base Station Signals

**DOI:** 10.1289/ehp.10870

**Published:** 2008-02

**Authors:** Andrew Cohen, George Carlo, Andy Davidson, Margaret White, Catarina Geoghan, Andrew Goldsworthy, Olle Johansson, Don Maisch, Eileen O’Connor

**Affiliations:** Powerwatch Sutton, Cambridgeshire, United Kingdom, E-mail: andrew@powerwatch.org.uk; Science and Public Policy Institute, Washington, DC; h.e.s.e.-UK, London, United Kingdom; Centre for Cognitive Science, University of Sussex, Brighton, United Kingdom; Imperial College London, London, United Kingdom; Department of Neuroscience, Karolinska Institute, Stockholm, Sweden; EMFacts Consultancy, Lindisfarne, Tasmania, Australia; EM-Radiation Research Trust, Exeter, United Kingdom

Electromagnetic hypersensitivity (EHS) is a potentially highly significant public health problem. Eltiti et al. (2007a) recently concluded that short-term exposure to a GSM (global system for mobile communication) base station–like signal did not affect well-being or physiological functions in individuals, and they dismissed a positive reaction to UMTS (universal mobile telecommunications system) as an artefact.

Eltiti et al. (2007a) stated that “[EHS] individuals are unable to detect the presence of rf-emf [radio frequency electromagnetic fields] under double-blind conditions.” We believe that this conclusion was erroneous, and that their data show that the EHS individuals reacted to both GSM and UMTS signals, and that this was not due to a nocebo effect.

Figure 1 presents their data [mean and SE from Table 2 (Eltiti et al. 2007a)] and clearly shows that the sensitive group, unlike the control group, was reacting to the exposure, with significant results in both the open provocation (for GSM and UMTS, note the sham; *p* < 0.0025) and the double-blind tests (for UMTS). The results for anxiety and arousal are very similar.

The sensitive group had higher initial levels of anxiety, tension, and arousal. Only a short time elapsed after arrival before testing started. Wever (1979) and others have reported that a period of a few days in a low-EMF environment are necessary before testing for EMF-related changes.

We are puzzled by the receiver operating characteristic (ROC) curves in Figure 2A (Eltiti et al. 2007a). The authors stated that the sensitive individuals were 55.2% correct, yet their curve was mostly below the 50% line. A more standard way of displaying the results would have been helpful. The sensitive group improved its on/off accuracy score after 50 min (55% to 60%), whereas the control group decreased (51% to 50%). The data for these double-blind tests (Fox E, personal communication) show that correct versus incorrect results were 60.6% (*p* < 0.005) for the sensitive group and 49.4% (not significant) for the control group.

Eltiti et al. (2007a) found a large and statistically significant (*p* < 0.001) higher skin conductance in the sensitive group (see their Table 5). Their conclusions do not highlight this difference between the two groups, which may be a key indicator of likelihood of individuals to experience EHS symptoms.

The EHS questionnaire devised by Eltiti et al. (2007b) was to be used for selecting the 132 most sensitive individuals. However, it was not used for this purpose because only 58 people with self-diagnosed EHS applied, and apparently no individuals were rejected because of a low score.

Are provocation studies appropriate for testing for EHS, where there is often a significant time-lag from start of exposure to the start of symptoms? Also, perseveration of symptoms due to physiological arousal caused by traveling to the laboratory is a likely confounder. Any study should be designed to take into account both of these potential problems.

Also, the use of Bonferroni corrections is contentious; uncorrected data should be shown along with corrected data.

The study (Eltiti et al. 2007a) required 66 individuals per group for a power 0.90 to detect a difference between real and sham exposure responses. The authors tested only 44 sensitive individuals under double-blind conditions, which reduced the power to about 0.7. We question the appropriateness of publishing such definite conclusions based on such data, especially with a high-profile media briefing.

Despite limitations, this study of Eltiti et al. (2007a) has produced positive results that support claims that EHS people can be affected by microwave transmissions from mobile phone base stations.

## Figures and Tables

**Figure 1:**
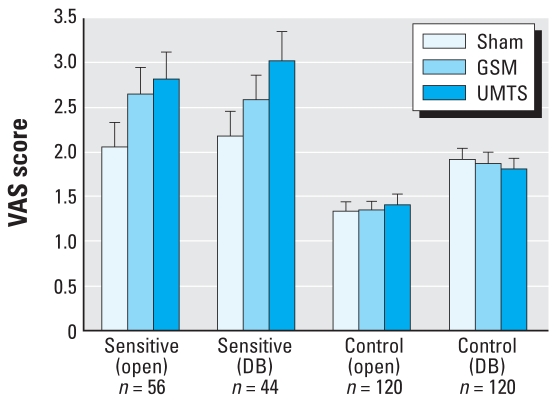
Tension (visual analog scale) scores (mean and SE) from Table 2 of Eltiti et al. (2007). DB, double-blind.
